# Effects of an exclusive human-milk diet in preterm neonates on early vascular aging risk factors (NEOVASC): study protocol for a multicentric, prospective, randomized, controlled, open, and parallel group clinical trial

**DOI:** 10.1186/s13063-021-05445-9

**Published:** 2021-07-31

**Authors:** Wolfgang Mitterer, Christoph Binder, Anya Blassnig-Ezeh, Lorenz Auer-Hackenberg, Angelika Berger, Burkhard Simma, Martin Wald, Martin Lee, Ursula Kiechl-Kohlendorfer

**Affiliations:** 1 VASCage GmbH, Research Centre on Vascular Ageing and Stroke, Innrain 66a, 6020 Innsbruck, Austria; 2 Department of Pediatrics II (Neonatology), Medical University of Innsbruck, Anichstraße 35, 6020 Innsbruck, Austria; 3 Department of Pediatrics and Adolescent Medicine, Comprehensive Center for Pediatrics, Division of Neonatology, Pediatric Intensive Care & Neuropediatrics, Medical University of Vienna, Währinger Gürtel 18-20, 1090 Vienna, Austria; 4 Department of Pediatrics, Academic Teaching Hospital Feldkirch, Carinagasse 47, 6800 Feldkirch, Austria; 5 Department of Pediatrics, Division of Neonatology, Paracelsus Medical University Salzburg, Strubergasse 21, 5020 Salzburg, Austria; 6 Fielding School of Public Health (UCLA), 650 Charles E. Young Dr. South, Los Angeles, CA 90095-1772 USA; 7 Prolacta Bioscience, 1800 Highland Ave, Duarte, CA 91010 USA

**Keywords:** Neonatology, Prematurity, Preterm, Human milk, Human-milk-based fortifier, Cardiovascular risk

## Abstract

**Background:**

Preterm birth accounts for approximately 11% of all livebirths globally. Due to improvements in perinatal care, more than 95% of these infants now survive into adulthood. Research has indicated a robust association between prematurity and increased cardiovascular risk factors and cardiovascular mortality. While the innate adverse effects of prematurity on these outcomes have been demonstrated, therapeutic strategies on the mitigation of these concerning developments are lacking. The primary objective of the NEOVASC clinical trial is therefore to investigate whether the administration of a prolonged exclusive human-milk diet in preterm infants is capable of alleviating the harmful effects of preterm birth on the early development of cardiovascular risk factors.

**Methods:**

The NEOVASC study is a multicentric, prospective, randomized, controlled, open, and parallel group clinical trial conducted in four Austrian tertiary neonatal care facilities. The purpose of the present trial is to investigate the effects of a prolonged exclusive human-milk-diet devoid of bovine-milk-based food components on cardiovascular and metabolic risk factors at 1, 2, and 5 years of corrected age. Primary outcomes include assessments of fasting blood glucose levels, blood pressure levels, and the distensibility of the descending aorta using validated echocardiographic protocols at 5 years of corrected age. The test group, which consists of 200 preterm infants, will therefore be compared to a control group of 100 term-born infants and a historical control group recruited previously.

**Discussion:**

Given the emerging implications of an increased cardiovascular risk profile in the potentially growing population of preterm infants, further research on the mitigation of long-term morbidities in formerly preterm infants is urgently warranted. Further optimizing preterm infants’ nutrition by removing bovine-milk-based food components may therefore be an interesting approach worth pursuing.

**Trial registration:**

ClinicalTrials.govNCT04413994. Registered on 4 June 2020.

**Supplementary Information:**

The online version contains supplementary material available at 10.1186/s13063-021-05445-9.

## Introduction

### Background

According to the World Health Organization (WHO), noncommunicable diseases (NCDs) are the world’s leading cause of death. Among NCDs, cardiovascular diseases (CVDs) are the most common ones, responsible for an estimated 17.8 million (31.8 %) deaths in 2017, and are therefore the single leading cause of global mortality [1, 2]. While family history and unhealthy lifestyle factors are undoubtedly associated with the development of CVDs, further research has identified early-life exposures as additional risk factors. Based on Barker’s “fetal origins of adult disease” hypothesis, adverse conditions in the intrauterine and perinatal environment may permanently alter physiologic circuits and organ function. Initially intended to aid survival, these adaptions may prove disadvantageous in the long-term by putting individuals at risk for the development of chronic cardiovascular and metabolic disorders [3, 4]. Recent evidence has suggested that preterm birth plays an important role in the fetal/neonatal programming of future morbidity [5] and mortality [6–9]. According to the most recent WHO estimates, preterm birth (< 37 weeks’ gestation) accounted for 10.6% of all livebirths worldwide with tendencies potentially increasing over time in middle- and high-income countries [10]. Due to advances in perinatal care [11, 12], the vast majority of these preterm infants now survive into adulthood [6–8]. However, several studies have shown an association between preterm birth and cardiovascular risk factors such as elevated blood pressure (BP) levels [13–16], hypertension [13, 17, 18], and type 1 or type 2 diabetes mellitus [19, 20], respectively. Compared with children born at term (≥ 37 weeks’ gestation), formerly preterm infants further display significantly reduced distensibility and increased stiffness of the descending abdominal aorta at preschool age [21], indicating initial signs of early vascular aging [22]. Mother’s milk is the optimal source of nutrition for all infants [23]. As for many preterm infants, the nutritional value of human milk is commonly enhanced by the use of fortifiers to meet their special needs. In cases with insufficient amounts of mother’s own or donor human milk, preterm formula is routinely used. Research has suggested potential benefits of an exclusive human-milk-based diet (i.e., the combination of mother’s own or donor human milk with human-milk-based fortifiers) on outcomes such as rates of necrotizing enterocolitis (NEC), duration of parenteral nutrition, and risk of late-onset sepsis and mortality when compared to a diet containing any bovine-milk-based products [24–27]. While the favorable effects of human milk on the development of the abovementioned cardiovascular and metabolic outcomes have been demonstrated despite methodological limitations [28, 29], no study has yet investigated the possible benefits of an exclusive human-milk-diet devoid of bovine-milk-based dietary components in preterm infants during the vulnerable preterm period on these very outcomes.

### The NEOVASC study

The NEOVASC study is a multicentric, prospective, randomized, controlled, open, and parallel group clinical trial conducted in four Austrian tertiary neonatal care facilities (Medical University of Innsbruck, Paracelsus Medical University Salzburg, Medical University of Vienna, Academic Teaching Hospital Feldkirch). The purpose of the present trial is to investigate the superiority of a prolonged (until 36 weeks’ gestation) exclusive human-milk-diet devoid of bovine-milk-based food components on cardiovascular and metabolic risk factors. By following formerly preterm infants until the corrected age of 5 years, we intend to facilitate research findings on the impact of specifically targeted early dietary interventions on long-term cardiovascular health. Previous research has shown that increases in BP [30, 31], alterations in glucose homeostasis [30, 32, 33], and vascular integrity [21] are visible even in early childhood following preterm birth. Given the inverse correlation between gestational age and adulthood diabetes [20] and hypertension [13, 18], respectively, we anticipate markedly significant results should our intervention in these preterm infants prove effective.

## Methods/design

### Study population and eligibility

#### Preterm-born infants

Extremely preterm infants with a birth weight between 500 g and 1250 g will be eligible to undergo randomization in the NEOVASC study. This particular weight range was chosen in accordance with previous clinical trials on exclusive human-milk diet [24–27]. Furthermore, written informed consent from the mother will be obtained in order to enroll participants. Exclusion criteria are birth weight < 500 g or > 1250 g, inborn errors of metabolism (e.g., galactosemia, phenylketonuria), not having been NPO or fed exclusive human-milk diet prior to enrollment, presence of major congenital malformation, presence of intestinal perforation or stage II NEC prior to enrollment, parents unwilling to sign informed consent, and lastly the inability to participate for any reason based on the study investigator’s judgment (e.g., unlikely to survive the study period).

#### Term-born infants

For the control group, term-born (≥ 37 weeks’ gestation) children with an adequate birth weight will be recruited at 5 years of age. Written informed consent will be obtained from the parent(s) or legal guardian(s) prior to enrollment. Exclusion criteria are acute and chronic illness or the unwillingness of the parent(s) to sign the informed consent form.

### Randomization, study groups, and interventions

**Fig. 1:**
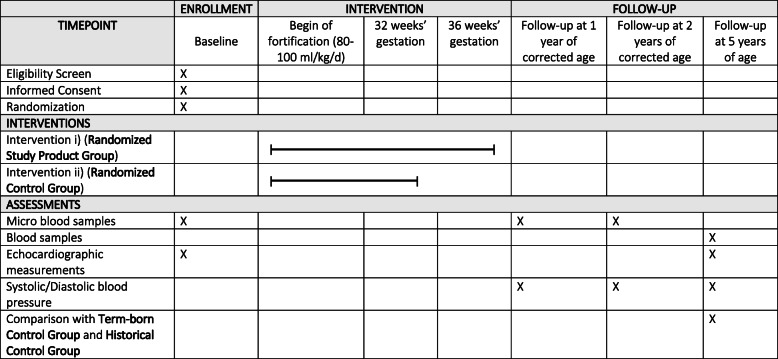
Standard Protocol Items: Recommendation for Interventional Trials (SPIRIT) figure of enrollment, intervention, and follow-up of preterm study participants

Randomization of preterm infants will be done electronically by the use of permuted blocks (block size = 4) within a stratified randomization. The allocation sequence will be generated by the study’s statistician and provided to the principal investigators in the form of a conventional unblinded randomization table. Stratification variables are the study site and birth weight (< 1000 g and 1000–1250 g). On the basis of ethical considerations, twins are always randomized into the same study group. Preterm neonates will be randomized to either (i) an exclusive human-milk-based diet composed of mother’s milk or pasteurized donor human milk in combination with a human-milk-based fortifier (Humavant^TM^ +4,+6; Prolacta Bioscience, Inc.) until 36 weeks of gestation (Randomized Study Product Group) or (ii) mother’s milk or pasteurized donor human-milk (as long as available) in combination with a human-milk-based fortifier (as long as human milk is available) until 32 weeks’ gestation and thereafter mother’s milk or pasteurized donor human milk with a bovine-milk-based fortifier or preterm formula (Randomized Control Group) in a 1:1 ratio. Feeding will be done by protocol in which fortification will begin when the infant is receiving 80–100 ml/kg/day of enteral nutrition. After 36 weeks’ gestation, human milk with a bovine-milk-based fortifier or preterm formula will be used in both groups. While blinding of study groups is always desirable in randomized studies, this is not possible for this study due to the nature of the intervention. As a control group, term-born age-matched infants will be included (Term-born Control Group). Infants within this control group will be recruited in kindergartens at 5 years of age. The results of the present study will further be compared to a group of very preterm infants enrolled for prior clinical studies at the Medical University of Innsbruck (Historical Control Group) [21, 30, 34]. See Figure S1 for a graphic display of the study design. The description of the overall study design is in agreement with the 2013 SPIRIT checklist (see Fig. 1).

### Study outcomes and follow-up

Outcomes include predictors and intermediate components of cardiovascular disease. Primary outcomes include assessments of the absolute difference in fasting blood glucose levels, blood pressure levels, and the distensibility of the descending aorta using validated echocardiographic protocols [21, 35] at 5 years of age. Secondary outcomes include assessments of fasting blood glucose levels and blood pressure levels at 1 and 2 years of corrected age as well as comparisons of body mass index and lipid profiles at 5 years of corrected age. Data on the clinical status of preterm infants in the study will be obtained at baseline during hospitalization as well as at routine follow-up visits at 1, 2, and 5 years of age, respectively. See Fig. 1 (SPIRIT figure) for a summary of all essential study procedures in the preterm study participants. Data from participants within the term-born control group will be obtained during a singular visit at 5 years of age. Data about birth and postnatal development from these participants will be taken from the mother-child-pass (official Austrian medical records on pregnancy, birth, and early childhood). In addition, cord blood, milk, stool, and urine samples will be obtained from preterm study participants at various points during the study. While milk samples will be used for analyses regarding osmolarity and nutritional values, all other samples will be stored at − 80° C for post-study analyses. As for preterm study participants from the Medical University of Vienna, body composition using body plethysmography (Pea Pod®) will further be measured at term-equivalent and 3 and 6 months of corrected age, respectively.

### Sample size and statistical analyses

A group size of 100 preterm infants per study arm is sufficient to detect a lowering in systolic blood pressure from 102 mmHg (Randomized Control Group) to 99 mmHg (Randomized Study Product Group), a reduction of fasting glucose levels from 84 to 78.5 mg/dl, and an increase in the distensibility of the descending aorta from 76 to 85 kPa^−1^ × 10^−3^, respectively. Based on previous experience, the principal investigators are confident to recruit the intended number of study participants within a recruitment period of 36 months. Each of these calculations was performed considering a power of 0.8 by the use of two-sample *T*-test for independent samples with homoscedastic variances. The effect size for this clinical trial was based on Cohen’s *D* assuming an effect size of 0.4. All statistical analyses will be performed on an intention-to-treat paradigm with a significance level of 0.05. Multiple outcomes will be handled using the Benjamini-Hochberg procedure for controlling the false discovery rate. Distensibility and stiffness index β of the descending abdominal aorta and lipid profiles will be compared between groups with two-sample *T*-tests or the Wilcoxon rank-sum test as a primary analysis at any given time point (looking at change from baseline). However, an adjusted analysis using a general linear model will incorporate the potential time trends in the results, as well as relevant covariates. Relationships between elasticity and stiffness, or lipid profiles and independent variables (sex, gestational age at birth, birth weight, ponderal index) will be studied by univariate and multivariate regression analysis. Missing data will be imputed using a multiple imputation with chained equations approach. In the event of repeated missing data over time, the simplistic last value carried forward approach may be used. However, in order to evaluate the data from a sensitivity analysis perspective, a secondary analysis on a per-protocol basis will be performed. This analysis will only consider participants who were correctly randomized and have complete data sets.

### Data management

Relevant data will be obtained using an electronic data capture system. After data acquisition is completed, a clean data file for the following statistical analyses will be established and stored. All investigators were trained with the electronic data system and regular monitoring visits are held to ensure proper data quality. Data management will be done by M.L. and W.M. Forwarding of patient-related data for further statistical analyses will only be done in pseudonymized or anonymized form. The code which enables the pseudonymized data to be allocated to a particular individual will be stored at the respective study centers. Following the completion of statistical analyses, data will be stored for 15 years in accordance with the “International Conference of Harmonization – Good Clinical Practice (ICH-GCP)” guidelines. All data will be handled in accordance with the EU General Data Protection Regulation and/or applicable national data protection regulations. Parents of study participants will be informed about their data protection rights in the informed consent form and written consent will be obtained.

### Safety

Preterm infants, and especially those born very and extremely premature, are an exceptionally vulnerable and often seriously ill patient population. Many adverse events (AEs) seen in this collective may be of serious nature. However, most of these AEs are expected to occur with no relationship to the intervention performed during the NEOVASC trial. In addition, the use of human milk and a human-milk-based fortifier (Humavant^TM^) in preterm infants is based on years of prior experience in clinical routine. Given that this is a nutritional study using a routinely used feeding product in the absence of pharmacological interventions, the investigators decided to move forward without an independent “Data Safety and Monitoring Board (DSMB)” prior to the study’s initiation and an interim analysis or stopping guidelines were not included in the current study design. AEs may be defined as medical NEC, confirmed late-onset sepsis as identified by positive blood cultures and feeding intolerance resulting in NPO status ≥ 24 h, change to infant formula, bloody stools, bilious emesis, or severe abdominal distension that results in a change with clinical nutritional management of the patient. Severe adverse events (SAEs) may be defined as death, surgical NEC, and lengthening of hospitalization. All SAEs and suspected unexpected serious adverse reactions (SUSARs) will be reported to the respective local ethics committee. If in the opinion of the attending physician there is a reasonable likelihood that any AE may have been causally associated with products from Prolacta Bioscience, Inc., or if the event results in a death regardless of the likelihood of association, Prolacta Bioscience, Inc., will additionally be notified and further clinical actions will be taken depending on the nature of the incident and the certainty of their causal relationship to the study product or study-specific procedures, respectively.

### Informed consent procedure and withdrawal

Expecting parents of potential participants will be invited by the respective principal investigators and/or explicitly permitted colleagues to enroll their preterm infants before or in a timely manner after delivery. Since additional data on the course of pregnancy will be gathered as well, written informed consent about the participation of preterm infants must be explicitly obtained from the infant’s mother. As for study participants in the term-born control group, written informed consent can be obtained from either parent or legal guardian. Subjects and their legal guardians have the right to withdraw consent fully or partially at any time and for any reason. All data until the point of withdrawal will be included in the final analysis. However, no further information will be collected. Protocol changes will be communicated to interested parties using the clinicaltrials.gov platform. Whenever protocol changes will affect study participants, informed consent will be obtained using an updated informed consent form.

### Ethics and trial registration

This protocol and all related documents have been approved by the ethics committee at each respective study site. The NEOVASC trial will be conducted in accordance with the Declaration of Helsinki in its most recent form and ICH-GCP guidelines. This study has been prospectively registered at clinicaltrials.gov (NCT04413994).

## Discussion

Given the abovementioned increasing preterm birth rates coupled with improvements in perinatal care and therefore low neonatal mortality rates, increases in long-term morbidities in formerly preterm infants are poised to become major public health challenges. This increased cardiovascular risk in formerly preterm infants may further strengthen the impact of CVDs as the world’s leading cause of death. A very recent Swedish population-based study of young male and female military conscripts found that formerly preterm infants were significantly more likely to exhibit elevated BP levels and an increased relative risk of systolic hypertension (defined as systolic BP ≥ 140 mmHg) in young adulthood when compared to those born at full- or post-term. The adjusted relative risk of developing systolic hypertension was 1.72 and 1.22 for female and male conscripts, respectively. For both women and men, an inverse relationship between gestational age and BP levels was observed [13]. These results corroborate previous findings in Swedish male military conscripts [36]. Another national cohort study from Sweden also found markedly increased risks of developing hypertension (defined as systolic BP ≥ 140 mmHg and/or diastolic BP ≥ 90 mmHg or taking antihypertensive medication) in young adulthood following preterm birth. This study also demonstrated an inverse correlation between gestational age and relative risk of developing hypertension. Therefore, formerly extremely preterm infants had a 1.8-fold risk of developing hypertension relative to infants born full-term (39–41 weeks’ gestation) [18]. These results find support in previously conducted meta-analyses on the association between preterm birth and elevated BP levels [14, 15]. With regard to the association between preterm birth and diabetes mellitus, a Swedish national cohort study found that the risk of developing type 1 or type 2 diabetes mellitus in young adulthood following preterm birth was 1.24 and 1.49, respectively when compared to infants born full-term. This study also found an inverse correlation between gestational age and risk of developing type 1 or type 2 diabetes mellitus. Extremely preterm birth was therefore associated with > 2-fold risks of developing both type 1 and type 2 diabetes mellitus [20]. These results find support in a previously conducted meta-analysis by Li et al. [19]. Both hypertension [37] and diabetes mellitus [38] are independent risk factors for the development of cardiovascular events. In addition to the abovementioned findings, a direct association between preterm birth and cardiovascular events later in life has been demonstrated. As for a CVD composite outcome consisting of ischemic heart disease, heart failure, cerebrovascular disease, and cardiovascular-related death, the risks of developing either one of these outcomes in the age group of 0–43 years were 1.57 and 5.68 for all preterm birth and extremely preterm birth, respectively, when compared to full-term birth [18]. Another study by Crump et al. [39] also found that preterm birth was significantly associated with ischemic heart disease in adulthood. Both studies [18, 19] further noticed an inverse correlation between gestational age and the development of cardiovascular events.

In conclusion, preterm birth has been shown to be associated with an adverse outcome in terms of cardiovascular health. While the benefits of an exclusive human-milk diet in preterm infants have been suggested for outcomes such as rates of NEC, duration of parenteral nutrition, risk of late-onset sepsis, and mortality [24–27], less is known about its effects on cardiovascular and metabolic risk factors. Given the emerging implications of an increased cardiovascular risk profile in the possibly steadily growing population of preterm infants, further research on the mitigation of long-term morbidities in formerly preterm infants is urgently warranted.

## Trial status

Protocol Version 4.7 (19 May 2020)

Trial status: Recruiting (first patient on 7 October 2020)

Study duration: Recruitment period of 36 months, follow-up until 5 years of age.

## Supplementary information

**Additional file 1: Figure S1.** Trial flow chart.

**Additional file 2.** SPIRIT 2013 Checklist: Recommended items to address in a clinical trial protocol and related documents.

## Abbreviations

AE: Adverse event
ALAT/ALT: Alanine aminotransferase
ASAT/AST: Aspartate transaminase
BP: Blood pressure
CRP: C-reactive protein
CVD: Cardiovascular disease
GOT: Glutamic oxaloacetic transaminase
GPT: Glutamate pyruvate transaminase
HDL: High-density lipoprotein
HOMA: Homeostasis model assessment
ICH-GCP: International Conference of Harmonization – Good Clinical Practice
IL-6: Interleukin-6
LDL: Low-density lipoprotein
NCD: Noncommunicable disease
NEC: Necrotizing enterocolitis
NT-proBNP: N-terminal prohormone of brain natriuretic peptide
NOP: Nil per os
SPIRIT: Standard Protocol Items: Recommendations for Interventional Trials
SUSAR: Suspected unexpected serious adverse reaction
TSH: Thyroid-stimulating hormone
WHO: World Health Organization
γ-GT: Gamma-glutamyltransferase
