# Supplementary figures and images for: Effects of an exclusive human-milk diet in preterm neonates on early vascular aging risk factors (NEOVASC): study protocol for a multicentric, prospective, randomized, controlled, open, and parallel group clinical trial
Source: Trials. 2021 Jul 31;22:509. doi: 10.1186/s13063-021-05445-9 (PMC8325296; doi:10.1186/s13063-021-05445-9)

E  
N  
R  
O  
L  
L  
M  
E  
N  
T

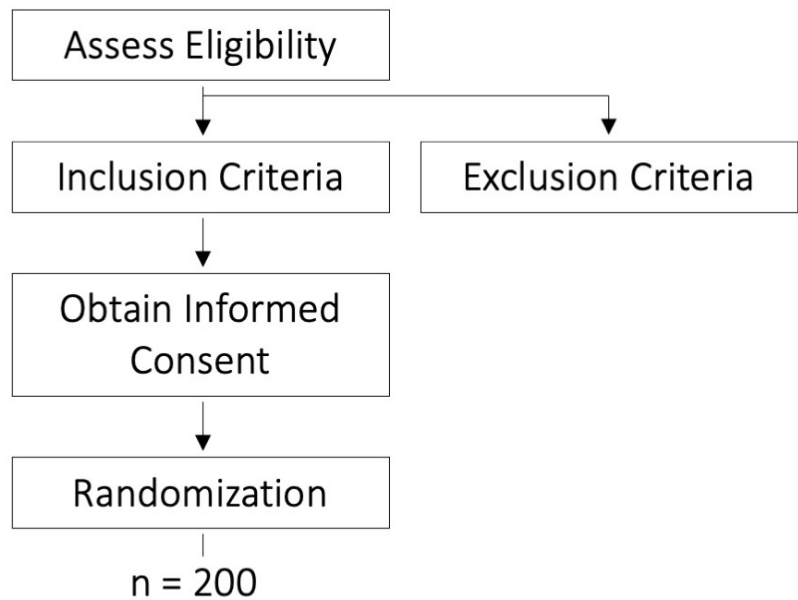

A  
L  
L  
O  
C  
A  
T  
I  
O  
N

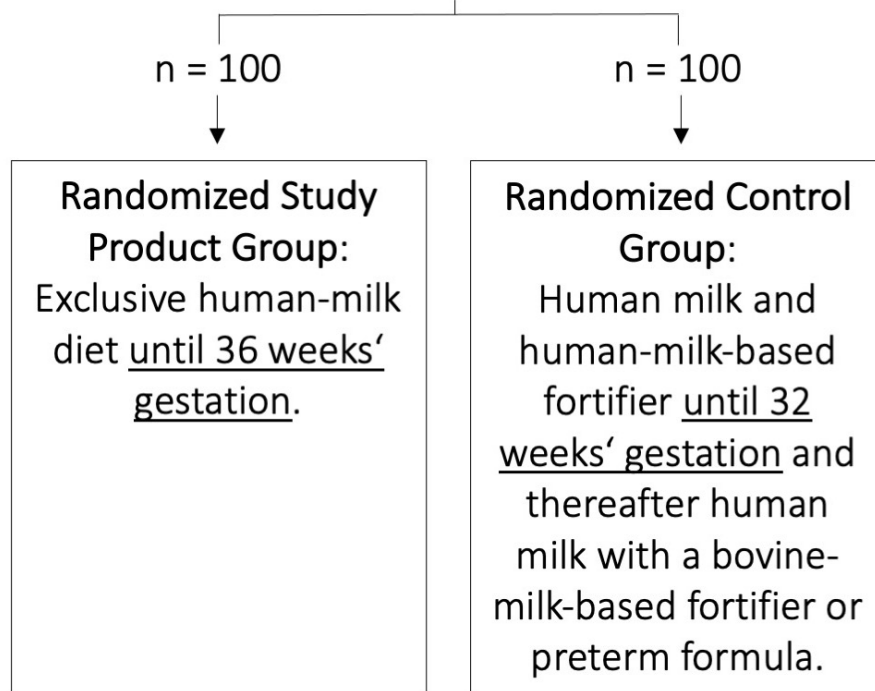

F  
O  
L  
L  
O  
W  
-  
U  
P

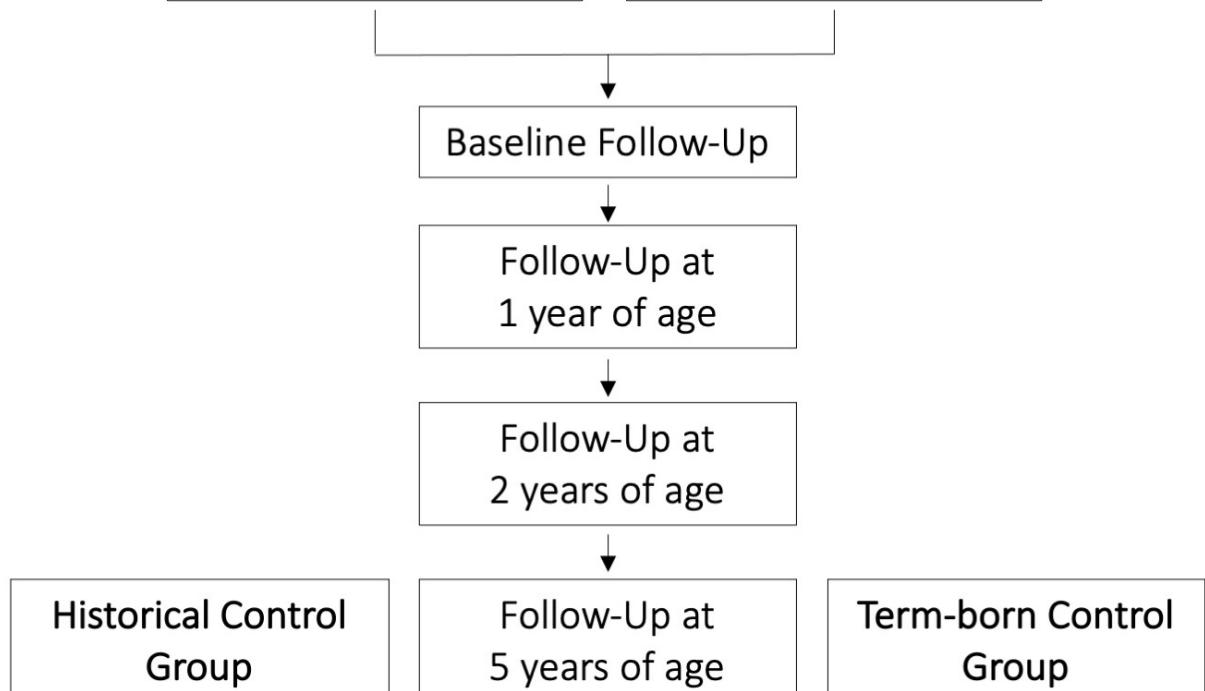

Supplement: Supplementary file 1 — Additional file 1: Figure S1. Trial flow chart. [file 13063_2021_5445_MOESM1_ESM.pdf]
